# An annotated dataset of tongue images supporting geriatric disease diagnosis

**DOI:** 10.1016/j.dib.2020.106153

**Published:** 2020-08-08

**Authors:** Dan Shi, Chunlei Tang, Suzanne V. Blackley, Liqin Wang, Jiahong Yang, Yanming He, Samuel I. Bennett, Yun Xiong, Xiao Shi, Li Zhou, David W. Bates

**Affiliations:** a Department of Geriatrics, Yueyang Hospital of Integrated Traditional Chinese Medicine and Western Medicine, Shanghai University of Traditional Chinese Medicine, Shanghai 200437, China; b Division of General Internal Medicine and Primary Care, Brigham and Women's Hospital, Boston, MA 02115, USA; c Clinical and Quality Analysis, Mass General Brigham, Somerville, MA 02145, USA; d Shanghai Shenkang Hospital Development Center, Shanghai 200041, China; e Department of Endocrinology, Yueyang Hospital of Integrated Traditional Chinese Medicine and Western Medicine, Shanghai University of Traditional Chinese Medicine, Shanghai 200437, China; f Shanghai Key Laboratory of Data Science, School of Computer science, Fudan University, Shanghai 201203, China

**Keywords:** Clinical documentation, tongue images, geriatric diseases

## Abstract

Hospitalized geriatric patients are a highly heterogeneous group often with variable diseases and conditions. Physicians, and geriatricians especially, are devoted to seeking non-invasive testing tools to support a timely, accurate diagnosis. Chinese tongue diagnosis, mainly based on the color and texture of the tongue, offers a unique solution. To develop a non-invasive assessment tool using machine learning in supporting a timely, accurate diagnosis in the elderly, we created an annotated dataset of 15% of 688 (=100) tongue images collected from hospitalized geriatric patients in a tertiary hospital in Shanghai, China. Images were captured via a light-field camera using CIELAB color space (to simulate human visual perception) and then were manually labeled by a panel of subject matter experts after chart reviewing patients’ clinical information documented in the hospital's information system. We expect that the dataset can assist in implementing a systematic means of conducting Chinese tongue diagnosis, predicting geriatric syndromes using tongue appearance, and even developing an mHealth application to provide individualized health suggestions for the elderly.

**Specifications Table**

**Table:** 

**Subject**	Aging
**Specific subject area**	Diagnosis – Image and text data analysis Hospitalized geriatric patients are a highly heterogeneous group often with variable diseases and conditions. Physicians, and geriatricians especially, are devoted to seeking non-invasive testing tools to support a timely, accurate diagnosis. The dataset can provide an objective test for Chinese tongue diagnosis, which is mainly based on the color and texture of the tongue.
**Type of data**	Free-text document Table Image Each patient has a folder with 1 face image, 1 tongue image, and 2 narrative documents. An additional summary formed by table is provided.
**How data were acquired**	We used a patented light-field camera (CN201520303463.5) called the intelligent mirror using CIELAB color space. Our data acquisition was handled in a standardized way (i.e., ensuring consistent sitting height and placement of the intelligent mirror) as much as possible.
**Data format**	The face and tongue images belong to raw data and were taken at 600 pixels per inch (about 42.3 *µ*m per pixel) and saved as a *.jpg with minimum compression (10% compression max). One narrative document is annotated and contains the parameters generated by the intelligent mirror when creating the face and tongue images, and the other contains the annotation results from the expert panel (e.g., vital signs, clinical imaging examination, and laboratory indicators).
**Parameters for data collection**	The study was conducted at a Chinese tertiary, comprehensive hospital. We recruited hospitalized subjects (excluding minority groups or other sensitive or disempowered populations) in the Geriatrics Department beginning in January 1, 2019. Images were captured via a light-field camera using CIELAB color space (to simulate the human visual perception) and then were manually labeled by a panel of subject matter experts after chart reviewing patients’ clinical information documented in the hospital's information system.
**Description of data collection**	Data acquisition and image annotation was conducted by subject matter experts including four fully credentialed senior-level physicians (i.e., associate chief physician and above), one resident, and two medical students. One medical student was in charge of data acquisition. The resident consolidated patients’ previous chronic medical history, clinical imaging examination, and laboratory indicators. One physician diagnosed patients’ constitutional types. Another physician gave a final admission diagnosis by considering the patient's constitution based on both traditional Chinese medicine and Western medicine. Constitutional types are based on TCM analysis and differentiation of pathological conditions in accordance with the eight principal syndromes, namely 八纲辨证, including yin and yang (阴阳), exterior and interior (表里), cold and heat (寒热), and hypofunction and hyperfunction (虚实). All the information from the free-text data labeling was documented digitally by one medical student in Chinese
	and translated into English. The treatment plan corresponding to the admission diagnosis was reviewed and annotated by the remaining two physicians. A total of 12 items must be merged into an annotated document, including various indices related to tongue diagnosis, physical or mental factors, clinicians’ observations, and more. To mitigate this, we used a previously designed algorithm to generate templates automatically. Under the *K*-means paradigm, our previously designed algorithm (1) embedded each annotated document into a vector representation for the first 200 patients, (2) partitioned those vectors into several (e.g., *K*=10) clusters, and (3) designated each cluster representative as a prototype template, or a vector of real annotated document closest to the centroid. For the remaining 468 patients, we used the specified prototype template to assist with the annotation.
**Data source location**	Shanghai, CHN Cambridge, MA, USA
**Data accessibility**	The data described in this Data note can be freely and openly accessed on the Harvard Dataverse. We also provided a data display at https://www.dataindustry.org/tongue. Repository name: Harvard Dataverse Data identification number: N/A Direct URL to data: https://doi.org/10.7910/DVN/COJZMQ Instructions for accessing these data: We uploaded 15% of 668 (=100) samples on the Harvard Dataverse. This dataset can be used as a training set. We are waiting for a Harvard Dataverse's new tool coming soon called DataTag that still allows sharing of sensitive data up to Harvard level 3 of data sensitivity. Before this, access to the full electronic copy will be provided based upon email request. The electronic dataset can be reached after having emailed to the corresponding author to get an approval, as a set of several split .zip files.
**Related research article**	C. Tang, J.M. Plasek, Y. Xiong, Z. Zhang, D.W. Bates, and L. Zhou. A Clustering Algorithm Based on Document Embedding to Identify Clinical Note Templates. Annals of Data Science, 2020.

**Value of the Data**

• The data is extensible, comparable, and compatible. Data collection processes are standardized to acquire data by considering the requirements and expectations of not only patients but also various researchers. Specifically, patients desire non-invasive, simple, and effective diagnostic tools. Clinicians are curious and sometimes want to collect data that doesn't exist in any pre-existing table of the database. Data analysts are interested in grouping data into categories that might not exactly fit the data. The dataset pursues at least three purposes. First, it covers almost all possible indicators of tongue diagnosis in Chinese and Western medicine and adds the content of face consultation additionally. Second, it aims to adopt the epidemiological method of investigation by (1) limiting the target population to Asia's elderly population aged 65 and over, and (2) scheduling the collection time as the first day of hospitalization. Thirdly, the data can be easily linked to data from different systems, such as CT (computerized tomography) scans or MRIs (magnetic resonance imaging) and clinical laboratory indicators, relying on more than 20 years of previous HIS (hospital information system) experience.
• The data is labeled by clinicians with rich clinical experience. A total of 16 physicians in the department of geriatrics participated in manually labeling the data with the admission diagnosis. Each patient's diagnosis is determined through a panel of subject matter experts. The data will be updated if the patient is readmitted to the hospital. The dataset meets the requirements for use as a training set and is suitable for artificial intelligence and machine learning. Some preliminary results are able to correct false medical information or misleading claims concerning tongue and face consultation on the Internet and social media.
• This continuously growing dataset (up to 688 patients) is new and original, and the data has not been published elsewhere.

## Data description

### Purpose of collection

Geriatric syndromes may be complicated and heterogeneous [1], and geriatric patients with multiple diagnoses are prone to treatment complications.

A unique, non-invasive approach to monitoring health [2] for millennia, tongue diagnosis purports that the tongue's color and texture are outer manifestations of the status of the internal organs [3] and provide insights into patient status in conditions like inflammation, infection, and endocrine disorders. Recently, tongue diagnosis has seen gradual acceptance in modern Western medicine, with the term “geographic tongue [4]” used to describe tongue discolorations or cracks accompanying illness. One case study of multiple systemic disorders published in the New England Journal of Medicine describes a patient's “smooth, shiny tongue [5].”

Applying machine learning to tongue images might provide useful diagnostic tools. Existing tongue appearance data is inadequate in both quality and quantity; therefore, we manually created an annotated tongue diagnosis dataset to support future work.

### Sample collection

The study was conducted at the Yueyang Integrated Traditional Chinese Medicine and Western Medicine Hospital, a tertiary, comprehensive hospital affiliated with the Shanghai University of Traditional Chinese Medicine. We recruited hospitalized subjects (excluding minority groups or other sensitive or disempowered populations) in the Geriatrics Department beginning in January 1, 2019. This study was approved by Yueyang's Institutional Review Board (IRB).

Since January 1, 2019, 668 adults were recruited to the study, of which 149 (22.3%) were male and 519 (77.7%) female. For each patient, two images of face and tongue were captured, and the associated free-text notes were collected and stored in a directory. We pulled unidentified patient ID (identification), age range, gender, weight and height, initial diagnosis, and admission and discharge dates, as well as previous chronic medical history from electronic medical records (EMRs) stored in Yueyang's hospital information system. We also collected vital signs, clinical imaging examination, and laboratory indicators during hospitalization.

Each patient has a folder with 1 face image, 1 tongue image, and 2 narrative documents. The face and tongue images were taken at 600 pixels per inch (about 42.3 *µ*m per pixel) and saved as a *.jpg with minimum compression (10% compression max). One narrative document contains the parameters generated by the intelligent mirror when creating the face and tongue images, and the other contains the annotation results from the expert panel (e.g., vital signs, clinical imaging examination, and laboratory indicators).

### Data acquisition and image annotation process

We ensured that the data covers as many indicators as possible, including the content of face consultation, in both Chinese and Western medicine. We used a patented light-field camera (CN201520303463.5) called the intelligent mirror using Commission on Illumination (CIE) L*a*b* color space (CIELAB) [6]. Patients’ health conditions may pose challenges during data collection. For example, some patients with cerebral infarction may have difficulty sticking out their tongue. Our data acquisition was handled in a standardized way (i.e., ensuring consistent sitting height and placement of the intelligent mirror) as much as possible.

Data acquisition and image annotation was conducted by subject matter experts including four fully credentialed senior-level physicians (i.e., associate chief physician and above), one resident, and two medical students. One medical student was in charge of data acquisition. The resident consolidated patients’ previous chronic medical history, clinical imaging examination, and laboratory indicators. One physician diagnosed patients’ constitutional types. Another physician gave a final admission diagnosis by considering the patient's constitution based on both traditional Chinese medicine (TCM) and Western medicine. Constitutional types are based on TCM analysis and differentiation of pathological conditions in accordance with the eight principal syndromes, namely 八纲辨证, including yin and yang (阴阳), exterior and interior (表里), cold and heat (寒热), and hypofunction and hyperfunction (虚实). All the information from the free-text data labeling was documented digitally by one medical student in Chinese and translated into English. The treatment plan corresponding to the admission diagnosis was reviewed and annotated by the remaining two physicians.

In the dataset, each patient had an individual folder consisted of 1 face image, 1 tongue image, and 2 narrative documents. The face and tongue images were taken at 600 pixels per inch (about 42.3 *µ*m per pixel) and saved as a *.jpg with minimum compression (10% compression max). Among two documents, one contains the parameters generated by the intelligent mirror when creating the face and tongue images, and the other document is the annotation results given by the expert panel related to the patient (e.g., vital signs, clinical imaging examination, and laboratory indicators). The two free-text documents were initially written in Chinese and then were translated into English by a medical student and approved by at least one of the experts.

**Fig. 1:**
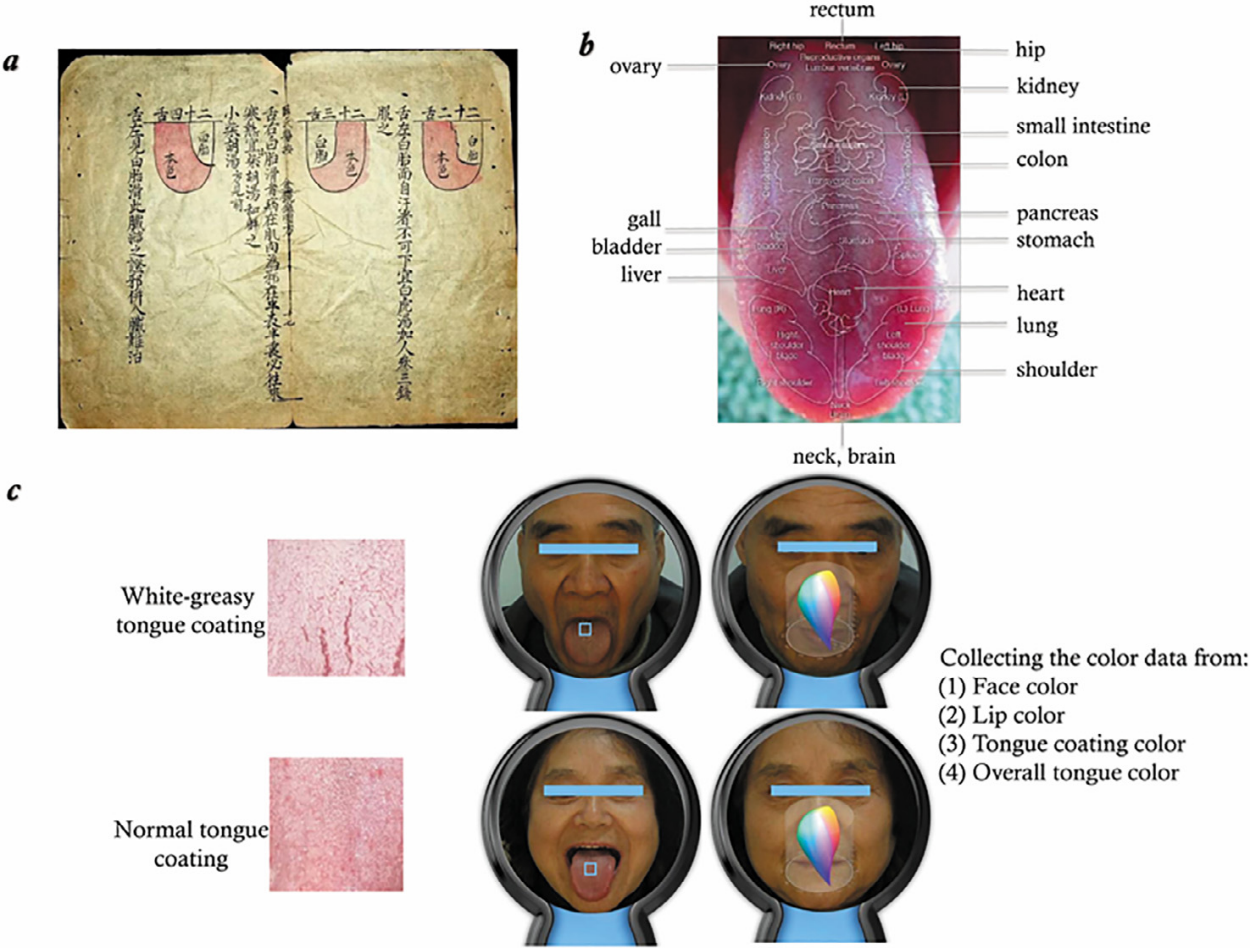
The principle of tongue data acquisition. *(a)* An ancient instruction for tongue diagnosis recorded in Ao-shi-shang-han-jin-jing-lu (i.e., 《敖氏伤寒金镜录》), a traditional Chinese medicine (TCM) book of 36 tongue illustrations compiled during the Yuan Dynasty of Ancient China; *(b)* Tongue appearance as an outer manifestation of the status of the human organ systems used as a guideline in the TCM; *(c)* Data acquisition process: *(1) t*ake images of 2 shots (i.e., face and tongue) of each patient, (2) capture colors via a light-field camera using CIELAB, a color space defined by the CIE in 1976.

Table 1, Table 2 shows the main parameters with their data type and references produced by the intelligent mirror, including the color of the *face, lip, tongue coating*, and *overall tongue*. For example, the color combination of cyan, red, yellow, white, and black were used to describe the color of the face according to the TCM literature.

**Table 1:** Overview of data files/data sets.

Label	Name of data file/data set	File types (file extension)	Data repository and identifier (DOI)
Data file 1	Sample of unlabeled data-1.csv	MS Excel file (.csv)	doi:10.7910/DVN/COJZMQ/NBIYEZ
Data file 2	Tongue_data_sample-1.xlsx	MS Excel file (.xlsx)	doi:10.7910/DVN/COJZMQ/AMXBHL
Data file 3	Consent_form	MS Docx files (.dcx)	doi:10.7910/DVN/COJZMQ/0PKYZ2
Data file 4	IRB_approval	PDF files (.pdf)	doi:10.7910/DVN/COJZMQ/HHISOT
Data file 5	Letter_of_support	PDF files (.pdf)	doi:10.7910/DVN/COJZMQ/X5MT7J
Image Data set	Replication data for: An annotated dataset of tongue images	Jpg files (.jpg)	doi.org/10.7910/DVN/COJZMQ

**Table 2:** Parameters produced by the intelligent mirror.

Items	Type of data	Description
Generation time	timestamp	YYYY-MM-DD
Face color	optical scope	3 (L, A, B value) as cyan, red, yellow, white, and black
Lip color	optical scope	3 (L, A, B value) as pale, pink, red, dark red, and purple
Tongue coating color	optical scope	3 (L, A, B value) as white and yellow
Overall tongue color	optical scope	3 (L, A, B value) as pale, pink, red, dark red, and purple

Table 3 indicates other items that were manually evaluated via expert judgment, including tongue shape (i.e., thick index), size (i.e., macroglossia index), the judgment of tooth marks or fissured tongue, and the degree of smoothness or shininess. We additionally documented the condition of each patient when taking photos in a text file located in the same directory as the face and tongue images, including the patient's sitting height and the distance between the patient and intelligent mirror.

**Table 3:** Manually annotated items.

Item	Type of data	Description	Category
Thick index	free text	normal or not (thick or fat)	expert judgment
Macroglossia index	free text	normal or not	expert judgment
Judgment of fissured tongue	free text	with or without cracks	expert judgment
Judgment of tooth marks	free text	with or without marks	expert judgment
Smooth or shiny index	free text	normal or not (smooth or shiny)	expert judgment
Constitutional types	free text	9 indicators (see Table 4)	physical factors
Scale for osteoporosis	questionnaire	primary and specific form	physical factors
Frailty assessment scale	questionnaire	SARC-F and FRAIL form	physical factors
Scale for geriatric depression	questionnaire	CGS short form	mental factors
Opinion for clinical imaging examination	free text	10 indicators (see Table 4)	clinical observations
Opinion for laboratory indicators	free text	20 indicators (see Table 4)	clinical observations
Additional information	free text	the patient's sitting height and the distance between the patient and intelligent mirror	other factors

Table 4 lists all the indicators used in the manually annotated documents.

**Table 4:** Indicators used in the manually annotated dataset.

Constitutional types	Clinical imaging examination	Laboratory indicators
Yin-yang harmony (平和质)	ECG (electrocardiogram)	Blood routine
Qi asthenia (气虚质)	Heart ultrasound	Urine routine
Yang asthenia (阳虚质)	Head magnetic resonance	Stool routine
Yin asthenia (阴虚质)	Chest CT (computed tomography) scan	Liver Function
Phlegm-dampness (痰湿质)	Abdominal ultrasound exam (liver, gallbladder, spleen, and pancreas)	Renal function
Blood stasis (血瘀质)	Renal ultrasound (kidneys, ureters, and bladder)	Blood Lipid
Dampness-heat (湿热质)	Thyroid ultrasound	B-type natriuretic peptide
Qi stagnation (气郁质)	Color doppler ultrasonography of the abdominal aorta (portal vein, the caudal vena cava, and their main abdominal branches and tributaries)	C-reactive protein
Special temperament (特禀质)	Venous ultrasound (lower extremity arteriovenous)	Erythrocyte sedimentation rate
	Carotid ultrasound	Blood electrolyte
		Carcinoembryonic antigen
		Thyroid function
		25 hydroxyvitamin D
		*β*-CTX
		Serum osteocalcin
		C-terminal (P1CP) propeptides of Type I collagen
		Thyroid peroxidase antibody
		Bone-derived alkaline phosphatase
		Glycated haemoglobin (HbA1c)
		Procalcitonin

## Experimental design, materials and methods

Manual annotation is a massive workload for physicians. A total of 12 items need to be merged into an annotated document, including various indexes related to tongue diagnosis, physical or mental factors, clinicians’ observations, and so forth. To help mitigate the workload associated with the process of manual annotation, we used a previously designed algorithm to generate templates automatically [7]. Under the *K*-means paradigm, our algorithm (1) embedded each annotated document into a 64-bit vector representation for the first 100 patients, (2) partitioned those vectors into several (e.g., *K*=10) clusters via the Hamming distance, and then (3) designated each cluster representative as a prototype template, which is a vector of real annotated document closest to the centroid. For the remaining 588 patients, we used one of the specified prototype templates (manually evaluated as the best) to assist with the annotation.

To our knowledge, this is the largest ongoing study to date to create a dataset of continuously collected, labeled tongue images. We envision that applying machine learning (such as deep neural networks) to tongue images might provide a useful diagnostic tool for geriatricians. This valuable labeled dataset may serve as training data in multiple scenarios in the future, such as describing Chinese tongue diagnosis systematically, predicting geriatric syndromes using tongue appearance, and even developing an mHealth application to provide individualized health suggestions based on tongue causes.

## Limitations

Our work was based on a single organization, Yueyang Integrated Traditional Chinese Medicine and Western Medicine Hospital, collecting from a single race in Asia, Chinese, and therefore might not be generalizable to other races and ethnicity categories.

The two free-text annotated documents may be misleading due to having been translated from their original language as well as algorithmic bias. These documents were initially written in Chinese, then translated into English by a medical student and approved by at least one of the experts.

## Ethics statement

We obtained trial approval at ClinicalTrials.gov. This project was approved by Yueyang Hospital's IRB without any “minority groups or other sensitive or disempowered populations.” All participants signed the consent form and agreed to share their data with face and tongue images.

The authors received full approval from all participants (without children or other “minority groups or other sensitive or disempowered populations.”). They understood that, (1) the information will be published without their names attached (but that full anonymity cannot be guaranteed), (2) the text and pictures or videos published in the article will be freely available on the internet and may be seen by the general public, and (3) the pictures, videos, and text may also appear on other websites or in print, and may be translated into other languages or used for commercial purposes.

## Declaration of Competing Interest

The authors declare that they have no known competing financial interests or personal relationships which have, or could be perceived to have, influenced the work reported in this article.

## Appendix Supplementary materials

Image, application 1
